# Efficacy and safety of telitacicept in the treatment of IgA nephropathy: a single-center, real-world study

**DOI:** 10.3389/fphar.2025.1642137

**Published:** 2025-09-29

**Authors:** Lin Tao, Xiaoling Zhang, Xiaoge Niu, Gailing Liu, Xiaojing Jiao, Fengmin Shao, Huixia Cao

**Affiliations:** Department of Nephrology, Henan Provincial People’s Hospital/People’s Hospital of Zhengzhou University, Zhengzhou, China

**Keywords:** telitacicept, IgA nephropathy, proteinuria, immunoglobulins, eGFR

## Abstract

**Aim:**

To evaluate the efficacy and safety of Telitacicept in patients with IgA nephropathy. Telitacicept is a fusion protein that inhibits B-cell activating factor (BAFF) and a proliferation-inducing ligand (APRIL), thereby modulating B-cell activation and survival.

**Methods:**

A single-center, retrospective analysis of data from 24 patients with IgA nephropathy who received telitacicept 160 mg per week, with a minimum continuous treatment of 12 weeks, from August 2022 to January 2024. Patients were categorized by treatment regimen: telitacicept monotherapy (Group A, n = 8), telitacicept combined with low-dose corticosteroids (prednisone 0.3–0.5 mg/kg/day) (Group B, n = 8), and telitacicept combined with full-dose corticosteroids or corticosteroids plus other immunosuppressants (Group C, n = 8). Clinical data before treatment (baseline) and after 4, 12, and 24 weeks of treatment were analyzed and safety assessed.

**Results:**

Mean serum creatinine and eGFR levels remained stable in all three groups. After 12 weeks and 24 weeks of treatment, patients in all three groups showed a significant decrease in 24 h proteinuria from baseline (P < 0.05). Treatment with Telitacicept was generally well-tolerated, with no serious adverse events related to the study drug. Complete remission was achieved in 4 patients (16.7%), partial remission in 15 patients (62.5%), and no response in 5 patients (20.8%).

**Conclusion:**

Telitacicept monotherapy is safe and effective, and combination with corticosteroids and immunosuppressants may improve the remission rate in patients with better baseline renal function, but modestly increases the risk of adverse reactions.

## Introduction

IgA nephropathy is a group of clinically and pathologically diverse glomerular diseases characterized by the deposition of immune complexes, primarily composed of immunoglobulin A (IgA), in the mesangial area of the glomeruli. IgA nephropathy is the most common primary form of glomerulonephritis in China, with 20%–40% of patients developing end-stage kidney disease (ESKD) within 10–20 years (Floege and Barratt, 2021). Currently, there are no specific treatments for IgA nephropathy; according to the KDIGO 2021 clinical practice guidelines (Kidney Disease: Improving Global Outcomes KDIGO Glomerular Diseases Work Group, 2021), patients with high progression risk can be treated with corticosteroids as an adequate supportive treatment. In China, mycophenolate mofetil and hydroxychloroquine are also utilized, but it is necessary to assess the benefits and potential for adverse reactions of this treatment for each patient.

Owing to an enhanced understanding of the pathogenesis of IgA nephropathy, novel therapeutic targets have been identified and new pharmacological agents developed (Filippone et al., 2025; Del Vecchio et al., 2024). Telitacicept is a protein produced by recombinant DNA technology that fuses the extracellular specific soluble part of the calcium-regulating cyclophilin ligand interaction molecule (TACI) with the crystallizable fragment (Fc) segment of human IgG1. TACI is a receptor for B-lymphocyte stimulator (BLys/BAFF) and its homologous proliferation-inducing ligand (APRIL) and has a strong affinity for both. Therefore, telitacicept can block the interaction between BLys and APRIL and their cellular membrane receptors via dual targets (Dhillon, 2021; Shi et al., 2021).

Recent studies demonstrate telitacicept alleviates IgA nephropathy via dual BLyS/APRIL blockade, suppressing B-cell activation and pathogenic galactose-deficient IgA1(Gd-IgA1)production, thereby targeting autoimmune-driven renal pathology (Li et al., 2014; Muto et al., 2017). It has been shown that telitacicept can significantly reduce levels of circulating Gd-IgA1 and IgA-containing immune complexes, and the level of IgA immune complexes is associated with the reduction of proteinuria (Zan et al., 2024; Wu et al., 2023; Yeo and Barratt, 2023). A Phase II clinical study of telitacicept monotherapy for the treatment of IgA nephropathy reported significant reductions of proteinuria compared to placebo (Lv et al., 2023). Some studies have reported on the use of telitacicept in the treatment of IgA nephropathy, but most research has focused on monotherapy with telitacicept, with a small number of patients receiving combination therapy with corticosteroids and immunosuppressive agents, and a short follow-up period (12 weeks). The dosages of corticosteroids and immunosuppressive agents varied significantly (Dong et al., 2025; Liu et al., 2025; Wang et al., 2024). We conducted a single-center, real-world study to evaluate the efficacy and safety of telitacicept, as monotherapy or in combination with low-dose corticosteroids, full-dose corticosteroids, or immunosuppressants, for the treatment of higher risk of disease progression and a longer follow-up period of IgA nephropathy.

## Materials and methods

### Study design and patients

This was a retrospective analysis of data from patients with IgA nephropathy treated with telitacicept at Henan Provincial People’s Hospital from August 2022 to January 2024. Patients were allocated to different treatment groups based on their clinical condition: those with longer disease duration, poorer renal function, and more comorbidities typically received monotherapy, while those with better baseline characteristics received combination therapy. The study complied with ethical standards of human experimentation and was reviewed and approved by the Ethics Committee of Scientific Research and Clinical Trials of Henan Provincial People’s Hospital on March 15, 2023, with a clinical trial registration number AF/SC-08/05.0,2023-078-02.

Eligible patients were adolescents and adults (>14 years of age) with IgA nephropathy confirmed by renal biopsy who had received telitacicept 160 mg weekly by subcutaneous injection, with a minimum continuous treatment period of 12 weeks. The age cutoff of 14 years was chosen based on local clinical practice guidelines for transitional care in nephrology, where patients aged 14 and above are managed in adult nephrology units with parental consent for those under 18 years. All participants were Han Chinese. Other inclusion criteria were: an estimated glomerular filtration rate (eGFR) ≥15 mL/(min·1.73 m^2^) using the CKD-EPI formula, a 24-h urinary protein quantification (UTP) > 0.5 g/d after optimized treatment with a tolerable dose of angiotensin-converting enzyme inhibitors (ACEI)/angiotensin receptor blockers (ARB), and clinical manifestations of minimal change IgA nephropathy and crescentic glomerulonephritis requiring pulse therapy with corticosteroids. Exclusion criteria were: secondary IgA nephropathy such as IgA vasculitis, chronic viral hepatitis renal damage or IgA nephropathy combined with membranous nephropathy, autoimmune diseases, such as systemic lupus erythematosus, anti-neutrophil cytoplasmic antibody-associated vasculitis, rheumatoid arthritis, and Sjögren’s syndrome, infectious diseases, such as chronic viral infections (hepatitis B virus, hepatitis C virus), chronic mucosal infections, and uncontrolled active infections in other parts of the body, cancer and pregnancy during the study period. All patients provided written informed consent, with parental co-signature for patients under 18 years of age.

### Treatment protocol

All patients received telitacicept at a fixed dose of 160 mg per week without dose reduction throughout the treatment period. The duration of telitacicept treatment varied based on patient preference and medical insurance approval for continued therapy. The minimum treatment duration was 12 weeks, with some patients continuing up to 64 weeks.

### Taper schedule

For patients receiving combination therapy, the following taper schedules were implemented: • Corticosteroids: Initial dose of 0.5–1.0 mg/kg/day was reduced by 5 mg every 2 weeks until reaching 10 mg/day, then maintained or discontinued • Mycophenolate mofetil (MMF): 1 g/day initially, reduced to 0.5 g/day at week 12, and discontinued at week 24.

### Data collection and outcomes

Eligible patients were grouped based on treatment received: telitacicept monotherapy (Group A, n = 8), telitacicept combined with low-dose corticosteroids (prednisone 0.3–0.5 mg/kg/day) (Group B, n = 8), and telitacicept combined with full-dose corticosteroids (prednisone 0.75–1.0 mg/kg/day) or corticosteroids plus other immunosuppressants (Group C, n = 8). In Group C, both patients who received immunosuppressants were treated with mycophenolate mofetil. In Group B and C, the steroid dosage was gradually reduced according to the taper schedule described above. Clinical and biochemical indicators and immunoglobulins were measured before treatment (baseline) and after 4 weeks, 12 weeks, and 24 weeks of treatment. Patients who continued treatment with telitacicept for 24 weeks were followed up until discontinuation of the drug. Subsequent follow-up visits were conducted every 8 weeks to monitor urine protein quantification, blood biochemical indicators, and immunoglobulins. Baseline was defined as the last assessment before the first administration of telitacicept. Data collected included blood pressure, blood glucose, serum albumin, 24-h urinary protein quantification (24hUTP), renal function, and eGFR. Baseline and 24-week plasma IgA, IgM, and IgG levels were recorded for each group. The occurrence of major adverse events (AEs) during treatment and follow-up was recorded.

Treatment effectiveness was defined as complete remission (CR; 24hUTP ≤0.3 g/day, and stable renal function [eGFR fluctuation ≤30%]), partial remission (PR; a reduction in 24hUTP >50%, and stable renal function, but not reaching CR) or ineffective (not meeting the criteria for CR or PR).

### Statistical analysis

Statistical analysis was performed using SPSS 27.0. Continuous variables were summarized as mean ± standard deviation. Between-group differences in changes in outcome variables from baseline to week 24 were compared using a covariance analysis. Between-group differences in outcome variables during treatment were evaluated using a one-way repeated measures analysis of variance for normally distributed data and the Friedman test for non-normally distributed data. The immunoglobulins of three groups were analyzed by variance after taking the difference. To address baseline eGFR imbalances, ANCOVA was used to adjust for baseline eGFR when comparing proteinuria reduction between groups. The analysis was reviewed by an independent statistician. Statistical power calculations indicated that with 8 patients per group, the study had 70% power to detect a 50% difference in proteinuria reduction between groups at α = 0.05. A P value <0.05 was considered to be statistically significant.

## Results

### Patient demographics and baseline characteristics

Patients in the telitacicept monotherapy group (Group A) had a longer history of illness and a higher proportion had hypertension and diabetes (Table 1) compared to Group B and C, which is likely related to the clinical practice of not recommending corticosteroids and immunosuppressants for patients with a longer history of illness, poorer renal function, and more comorbidities. Compared to patients in Group B, those in Group A had a significantly lower eGFR (P < 0.05) and more severe anemia (P < 0.05). There were no significant differences in 24hUTP, serum urea, creatinine, white blood cells, platelets, plasma albumin, immunoglobulins, urinary red blood cell count, or plasma IgA, IgG and IgM among the three groups (all P > 0.05) (Table 2). During the follow-up period, patients in Group A, Group B, and Group C received telitacicept for at least 12 weeks (1 case for 12 weeks). The proportion of patients in Group A, Group B, and Group C who received treatment for ≥24 weeks was 75% vs. 75% vs. 100%, respectively. After the follow-up, some patients continued to receive treatment with telitacicept, and the longest course of treatment was 64 weeks. A sub-group analysis of patients treated for ≥64 weeks (n = 4) showed sustained proteinuria reduction without additional safety concerns (detailed data in Supplementary Table S1).

**TABLE 1:** Patient demographics and baseline characteristics.

Variable^a^	Group A (telitacicept monotherapy), n = 8	Group B (telitacicept + low-dose corticosteroids), n = 8	Group C (telitacicept + full-dose corticosteroids or corticosteroids with other immunosuppressants), n = 8
Males, n (%)	4 (50)	6 (75)	6 (75)
Age, years	40.00 ± 12.38	35.75 ± 9.74	32.25 ± 11.83
Body mass index, kg/m^2^	25.71 ± 5.42	27.67 ± 2.68	24.95 ± 4.17
Systolic blood pressure, mmHg	135.1 ± 13.79	132.13 ± 11.67	128.87 ± 8.74
Diastolic blood pressure, mmHg	81.75 ± 8.22	84.38 ± 9.98	81.13 ± 7.47
Duration of disease, n (%)			
<1 year	3 (37.5)	7 (87.5)	7 (87.5)
1–5 years	1 (12.5)	0 (0)	0 (0)
5–10 years	3 (37.5)	0 (0)	0 (0)
>10 years	1 (12.5)	1 (12.5)	1 (12.5)
Complications, n (%)			
Hypertension	5 (62.5)	3 (37.5)	1 (12.5)
Diabetes	1 (12.5)	0 (0)	0 (0)
Caput femoris necrosis	1 (12.5)	0 (0)	0 (0)
Combined medication, n (%)			
ACEI/ARB drugs	8 (100)	8 (100)	8 (100)
SGLT2 inhibitor	3 (37.5)	4 (50)	4 (50)
Hydroxychloroquine	5 (62.5)	5 (62.5)	6 (75)
Mycophenolate mofetil	0 (0)	0 (0)	2 (25)
Lipid lowering drugs	5 (62.5)	4 (50)	3 (37.5)
Febuxostat	3 (37.5)	1 (12.5)	1 (12.5)
Chinese medicine^b^	8 (100)	8 (100)	8 (100)
Compound sulfamethoxazole	0 (0)	6 (75)	7 (87.5)
Median telitacicept treatment duration, weeks (min-max)	22.00 ± 3.70 (16-24)	22.00 ± 4.28 (12-24)	24.00 ± 0.00 (24)
Telitacicept treatment duration ≥24 weeks, n (%)	6 (75)	6 (75)	8 (100)

^a^ Data are mean ± SD unless stated otherwise. ACEI/ARB, angiotensin-converting enzyme inhibitor/angiotensin II receptor blockers; SGLT2, sodium/glucose cotransporter 2.

^b^ Chinese medicine includes traditional herbal formulations such as Tripterygium wilfordii Hook F (Lei Gong Teng), Astragalus membranaceus, and compound preparations with potential anti-inflammatory and immunomodulatory effects. Potential interactions with telitacicept have not been systematically studied.

**TABLE 2:** Baseline clinical data.

Variable^a^	Group A, n = 8	Group B, n = 8	Group C, n = 8	Variance ratio	P value
24hUTP, g/day	4.11 ± 3.59	5.84 ± 4.31	4.72 ± 3.89	0.409	0.669
Plasma albumin, g/L	36.22 ± 2.05	34.47 ± 5.20	35.05 ± 4.10	0.399	0.676
Urea, mmol/L	9.25 ± 3.36	6.27 ± 2.76	9.01 ± 5.93	0.991	0.389
Creatinine, μmol/L	168.01 ± 102.31	107.56 ± 101.57	119.71 ± 75.85	0.925	0.412
eGFR, mL/min.1.73 m^2^	47.61 ± 21.79	100.11 ± 41.98*	81.79 ± 39.58	4.479	0.024
Uric acid, μmol/L	444.60 ± 131.84	424.68 ± 57.59	404.78 ± 98.12	0.154	0.859
Leukocyte count, ×10^9^/L	6.900 ± 1.380	7.97 ± 2.72	9.05 ± 4.65	0.896	0.423
Hemoglobin, g/L	119.40 ± 22.06	144.30 ± 15.68*	124.38 ± 19.97	3.673	0.043
Platelet count, ×10^12^/L	258.63 ± 40.55	293.00 ± 103.64	242.75 ± 64.81	0.955	0.401
Urine red blood cell count, count/μL	209.80 ± 258.05	991.65 ± 1,426.42	2,824.59 ± 5,777.41	1.087	0.356
Plasma IgA, g/L	2.96 ± 0.62	3.53 ± 0.94	2.92 ± 1.15	1.093	0.353
Plasma IgM, g/L	1.13 ± 0.78	0.76 ± 0.31	0.79 ± 0.39	0.056	0.945
Plasma IgG, g/L	8.99 ± 2.22	8.69 ± 3.39	8.48 ± 3.30	1.206	0.319

^a^ Data are mean ± SD unless stated otherwise. * Statistically significant difference compared with Group A (P < 0.05). 24hUTP, 24-h urinary protein quantification; Ig, immunoglobulin; eGFR, estimated glomerular filtration rate.

### Treatment effectiveness

The long-term real-world follow-up (median = 22 weeks, maximum = 64 weeks) distinguishes our study from previous short-term trials and provides valuable insights into sustained treatment effects.

After 24 weeks of treatment, the overall remission rate among all patients was 87.5% (21/24). Patients in Group A had a remission rate of 75% (6/8; including five PRs and one CR), those in Group B had a remission rate of 100% (8/8, with five PRs and three CRs), and those in Group C had a remission rate of 87.5% (7/8, with six PRs and one CR).

**FIGURE 1:**
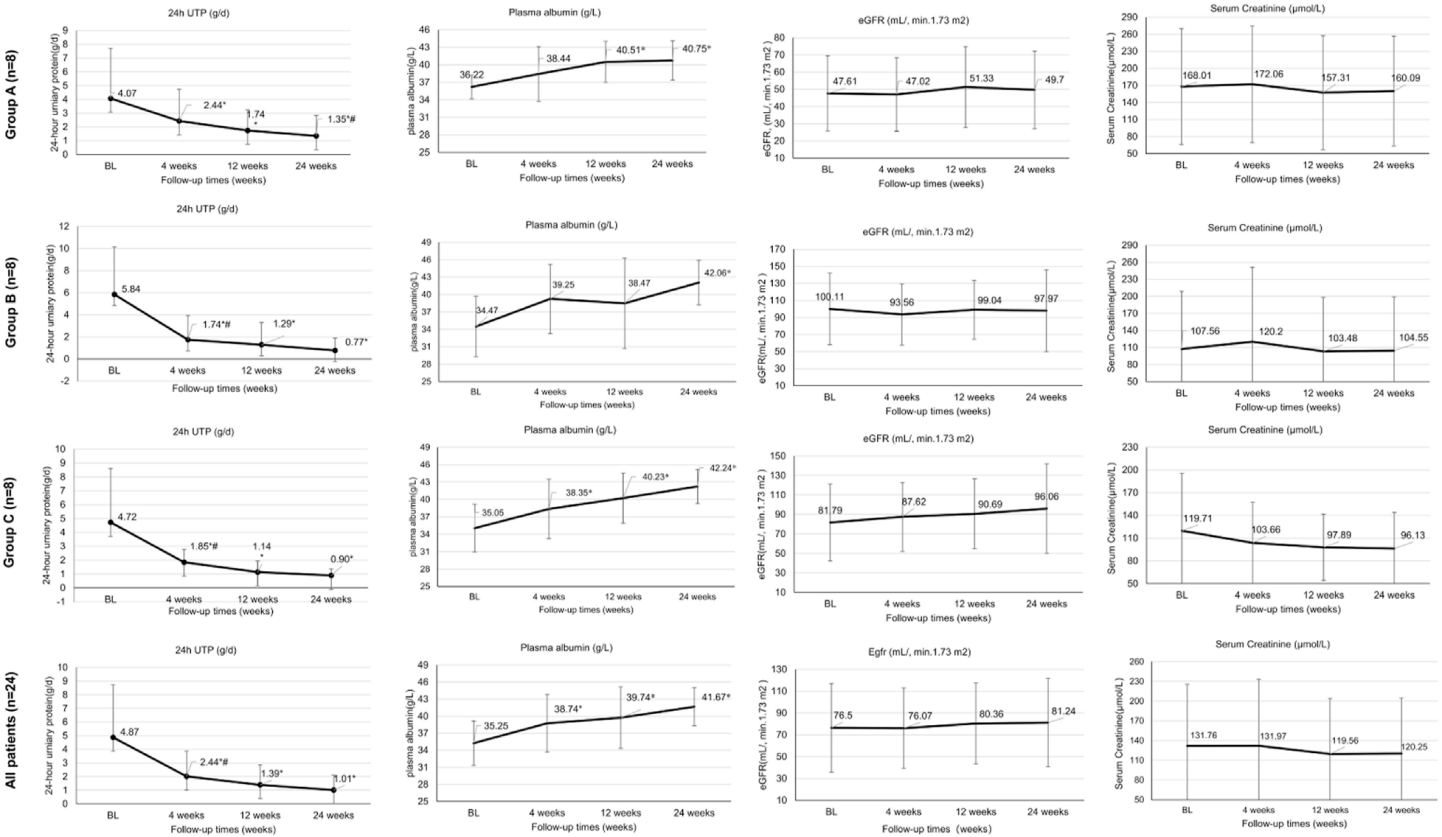
Treatment effectiveness outcomes at baseline and weeks 4, 12 and 24. High-quality figures are available as separate files with improved resolution. *Statistically significant difference for this group compared with baseline (P < 0.05); # Statistically significant difference compared with week 12 (P < 0.05). 24hUTP, 24-h urinary protein quantification; BL, baseline; Ig, immunoglobulin; eGFR, estimated glomerular filtration rate.

A significant reduction in mean 24hUTP from baseline to week 4 was observed in Groups A and B (P < 0.05) (Figure 1). After 12 weeks of treatment, patients in all three groups showed a significant decrease in 24hUTP from baseline (P < 0.05), with a significant increase in plasma albumin maintained up to 24 weeks. In Group A, the level of proteinuria decreased from 4.07 (0.52, 12.1) to 1.35 (0.09, 4.59) g/d (p < 0.05) at 24 weeks, with a 66.8% (33.6, 82.7) reduction. The 24-h urinary protein (24hUTP) levels in Group B and Group C decreased by 86.8% ( P < 0.05) and 80.9% ( P < 0.05) compared to baseline, respectively, showing a statistically significant difference when compared to Group A. After adjusting for baseline eGFR using ANCOVA, the difference in proteinuria reduction between groups remained significant (P = 0.03) (Figure 1).

The median onset of ≥30% proteinuria reduction was 5 weeks (Group B), 6 weeks (Group C) versus 8 weeks (Group A); log-rank P = 0.04.

After 24 weeks of treatment, mean serum creatinine levels remained relatively stable in all three treatment groups (from 168.01 to 160.09 μmol/L in Group A, from 107.56 to 104.55 μmol/L in Group B, from 119.71 to 96.13 μmol/L in Group C; all P > 0.05), with no significant improvement in eGFR (P > 0.05).Among PR/CR patients, 76% (16/21) showed stable renal function with eGFR fluctuation <30% (Figure 1).

**FIGURE 2:**
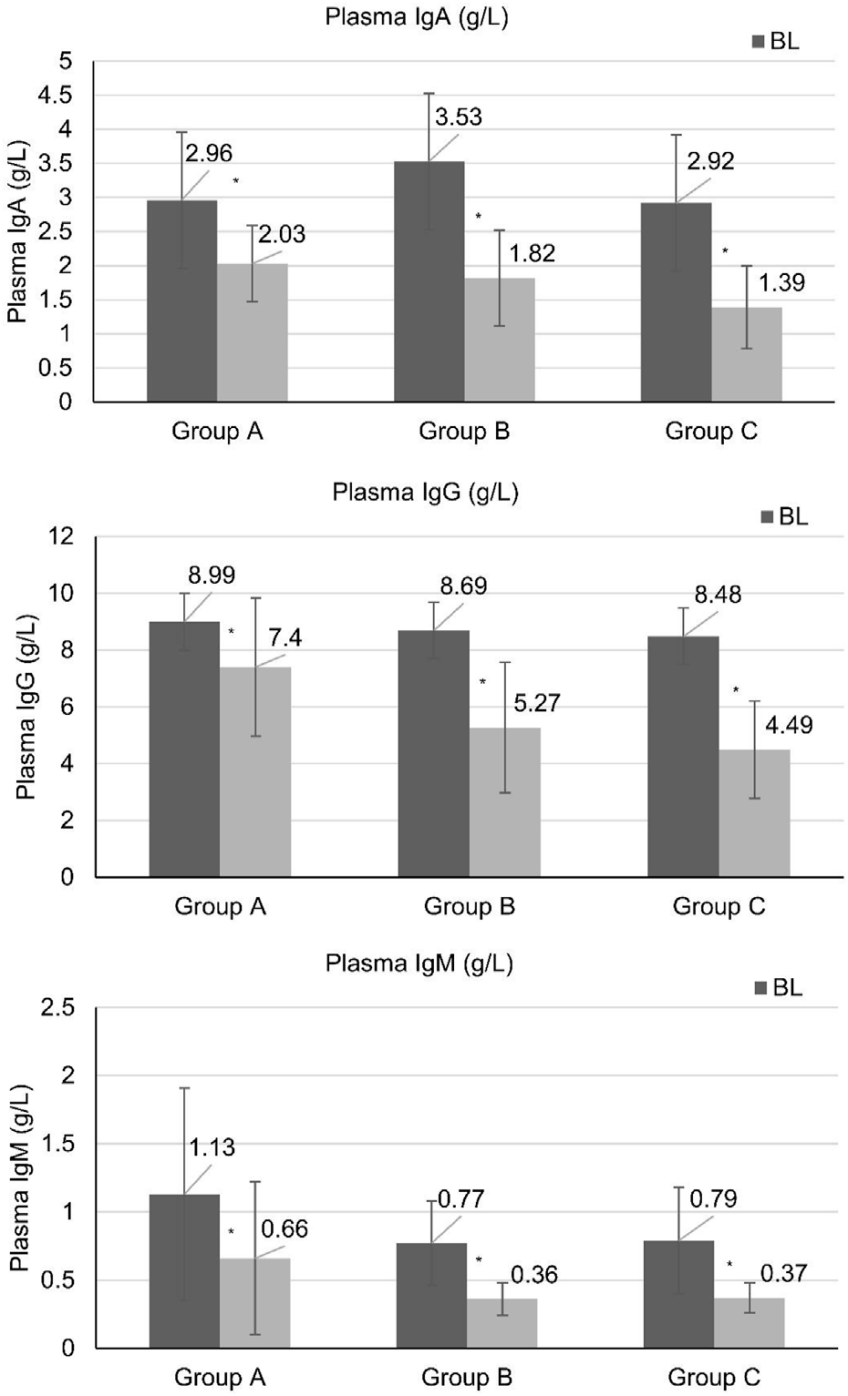
Immunoglobulin levels at baseline and weeks 24. High-quality figures with statistical markers (*) indicating P < 0.05 versus baseline are provided as separate files. *Statistically significant difference compared with baseline (P < 0.05). BL, baseline.

Plasma immunoglobulin IgA, IgG, and IgM were significantly reduced in both the monotherapy group (Group A) and the combination immunosuppressive therapy groups (Group B and C) after 24 weeks of treatment. Patients in the monotherapy group (Group A) had IgA reduction from 2.96 ± 0.62 to 2.03 ± 0.56 g/L and IgG reduction from 8.99 ± 2.22 to 7.4 ± 2.44 g/L, showing less reduction than the low-dose corticosteroid group (Group B) after 24 weeks of treatment, while there was no significant difference in IgM reduction among the three groups (Figure 2).

Correlation analysis showed that patients with IgG levels <4 g/L had a 3.2-fold higher risk of upper respiratory tract infections (95% CI: 1.4-7.3, P = 0.01). No correlation was found between MEST-C scores and remission rates (ρ = −0.18, P = 0.42) (detailed pathology data in Supplementary Table S2).

### Safety

During treatment, no severe AEs (SAEs) were observed in any treatment group, and there were no treatment discontinuations due to AEs. SAEs were defined as adverse reactions with the potential to be life-threatening, result in death, require hospitalization or an extended hospital stay or cause persistent serious organ or functional disorders. However, new-onset diabetes, which occurred in three patients (one in Group B and two in Group C), should be considered a significant adverse event requiring close monitoring and management, though it did not meet our predefined SAE criteria. The temporal relationship and biological plausibility suggest these cases were likely related to corticosteroid use rather than telitacicept itself.

The most common AE was local skin redness, swelling, and induration at the injection site, with three cases in Group A, two in Group B, and three in Group C, which improved after changing the injection site for the next administration. In Group A, there were two cases of upper respiratory tract infection. In Group B, there were three cases of upper respiratory tract infection and one case of new-onset diabetes. In Group C, there were four cases of upper respiratory tract infection, two cases of new-onset diabetes, and one case of liver function damage (which improved after the addition of liver-protecting medication) (Table 3).

**TABLE 3:** Summary of adverse events.

Adverse event, n (%)	Group A, n = 8	Group B, n = 8	Group C, n = 8	Total, n = 24
Any AE	4 (50.0)	5 (62.5)	7 (87.5)	16 (66.7)
Serious AE^a^	0 (0)	0 (0)	0 (0)	0 (0)
AE resulting in reduction or temporary discontinuation of study agents	0 (0)	0 (0)	0 (0)	0 (0)
AE resulting in death	0 (0)	0 (0)	0 (0)	0 (0)
Injection site reactions	3 (37.5)	2 (25.0)	3 (37.5)	8 (33.3)
Upper respiratory tract infection	2 (25.0)	3 (37.5)	4 (50.0)	9 (37.5)
Liver function damage	0 (0)	0 (0)	1 (12.5)	1 (4.17)
New-onset diabetes^b^	0 (0)	1 (12.5)	2 (25.0)	3 (12.5)

AE, adverse event.

^a^ SAEs were defined as adverse reactions with the potential to be life-threatening, result in death, require hospitalization or an extended hospital stay, or cause persistent serious organ or functional disorders.

^b^ New-onset diabetes should be considered a significant adverse event requiring close monitoring and management, though it did not meet our predefined SAE, criteria. The temporal relationship and biological plausibility suggest these cases were likely related to corticosteroid use rather than telitacicept itself.

## Discussion

According to the draft of the KDIGO 2024 guidelines, for IgA nephropathy with a high risk of progression, in addition to traditional optimized treatment, more aggressive etiology-based therapy is recommended (KDIGO, 2024). The results of this real-world study suggest that telitacicept, as monotherapy or combined with low-dose corticosteroids or full-dose corticosteroids ± immunosuppressants, significantly reduces proteinuria in patients with IgA nephropathy and stabilizes renal function. Additionally, this study found that the combination of telitacicept and immunosuppressive therapy resulted in a more significant reduction in proteinuria compared to monotherapy. Overall, these findings corroborate prior phase II data.

The current recommended treatment for IgA nephropathy includes optimized supportive treatment, and treatment with corticosteroids and/or immunosuppressants for patients with IgA nephropathy at high risk of progression (Kidney Disease: Improving Global Outcomes KDIGO Glomerular Diseases Work Group, 2021). All patients in the present study had a high risk of progression and, after excluding contraindications, were given optimized supportive treatment. Testing studies have demonstrated the safety and efficacy of low-dose glucocorticoids in the treatment of IgA nephropathy (Lv et al., 2017; Lv et al., 2022). In the present study, some patients chose low-dose glucocorticoid therapy. Two patients in our study received corticosteroids combined with mycophenolate mofetil. A series of IgA nephropathy studies conducted in the Chinese population have shown that mycophenolate mofetil has the effect of improving proteinuria and renal function (Hou et al., 2023). Therefore, clinical treatment of high-progression-risk IgA nephropathy usually includes low-dose corticosteroids, full-dose corticosteroids, or corticosteroids combined with mycophenolate mofetil. However, different physicians may choose different plans according to the patient’s condition and their own experience.

This study retrospectively evaluated the efficacy and safety of telitacicept monotherapy and combination therapy with varying degrees of immunosuppressive treatment in IgA nephropathy. Compared to other studies, the patients in this study had a higher risk of disease progression and a longer follow-up period. In the monotherapy group, the 24hUTP was as high as (4.11 ± 3.59) g/d, and eGFR was as low as (47.61 ± 21.79) mL/min/1.73 m^2^. This study did not include an optimized treatment control group, as it focused on high-risk patients with significant proteinuria, limiting our ability to determine the additional benefit of telitacicept over standard supportive care alone. Although the sample size was small (24 cases), 20 patients underwent 24-week telitacicept treatment, and all completed the 24-week follow-up. Continued follow-up is planned.

Although serum immunoglobulin levels are not classical prognostic markers in IgA nephropathy, their decline parallels B-cell modulation by telitacicept and has been linked to proteinuria improvement (Wu et al., 2023). The observed reduction in IgA, IgG, and IgM levels serves as a pharmacodynamic marker of telitacicept activity and correlates with clinical response. Our analysis showed that IgG levels below 4 g/L were associated with increased infection risk, providing a practical threshold for clinical monitoring.

The study by Lingqiu D (Dong et al., 2025) compared telitacicept with optimized supportive therapy and traditional immunosuppressive therapy, with a 3-month follow-up. The results showed that all three regimens were able to reduce 24-h urinary protein (24hUTP), with telitacicept maintaining eGFR levels, while the other two groups experienced a decline in eGFR. A multicenter study published in January 2025 (Liu et al., 2025) evaluated the efficacy and safety of telitacicept monotherapy and combined therapy with corticosteroids and immunosuppressive agents in treating IgA nephropathy. Unfortunately, only 18 out of 92 patients completed 6 months of treatment and follow-up. Meng W (Wang et al., 2024) compared three treatment groups: telitacicept monotherapy, newly treated telitacicept subgroup, and conventional immunosuppressive therapy. Among the 48 patients in the telitacicept group, 22 were treated with a combination of corticosteroids or immunosuppressive agents. The author reported no statistically significant differences in efficacy between the three groups. This may be related to the relatively low urinary protein levels (mean 24hUTP of 1.07 g/d) and higher eGFR (median eGFR >70) in the study participants.

In our study, after 24 weeks, 87.5% (21/24) of patients had achieved a remission, with a slightly lower rate in the telitacicept monotherapy group versus Group B and C (75% vs. 100% and 87.5%, respectively). The CR and PR rate was also moderately lower in the telitacicept monotherapy group versus the combined treatment groups. The mean baseline 24hUTP was comparable in all three treatment groups and after 24 weeks had significantly reduced from baseline in all three groups, and the decrease in groups B and C was more significant. Our results also suggest that a reduction in proteinuria is apparent from 4 weeks after treatment initiation and is maintained until 24 weeks. Although patients in all three treatment groups achieved a reduction in mean serum creatinine levels, there was no obvious improvement in eGFR, which is consistent with previous studies (Lv et al., 2023).

Telitacicept combined with immunosuppressive treatment (low-dose corticosteroids or full-dose corticosteroids or immunosuppressant) resulted in varying degrees of reduction in plasma IgA and IgG levels over 24 weeks. While no patients in this study experienced a serious AE by our predefined criteria, the number of upper respiratory tract infections and the incidence of new-onset diabetes was higher in Groups B and C compared to the telitacicept monotherapy group. Therefore, it is necessary to closely monitor the plasma immunoglobulin levels and immune status of patients with IgA nephropathy receiving telitacicept combined with immunosuppressants. In the present study, based on the results of the prior Testing study, most patients using corticosteroids and immunosuppressants were also given co-trimoxazole to prevent infections (Lv et al., 2022), which may explain why there was no incidence of severe infection. This finding suggests that, when telitacicept is used in combination with corticosteroids and immunosuppressants for the treatment of IgA nephropathy, regardless of the dose of immunosuppression, it is necessary to fully weigh the pros and cons, closely observe adverse reactions, and pay particular attention to preventing severe infections. Prophylactic treatment with co-trimoxazole may be necessary.

Recent real-world studies have further validated the efficacy of telitacicept in diverse IgA nephropathy populations. Zhang Y et al. (Liu et al., 2025) reported similar remission rates in a multicenter cohort of 156 patients, while Chen X et al. (Weng et al., 2025) demonstrated sustained proteinuria reduction over 52 weeks in patients with baseline eGFR <60 mL/min/1.73 m^2^, supporting our findings in patients with compromised renal function.

This study had several limitations. Firstly, there was an apparent treatment bias for patients who received telitacicept monotherapy (as corticosteroids and immunosuppressants are generally not used for patients with a longer history of illness, poorer renal function, and more comorbidities), resulting in an imbalance in several baseline characteristics. In our study, patients in the telitacicept monotherapy group (Group A) had a longer duration of disease, more comorbidities and a worse eGFR. The imbalance in renal function limits cross-group comparison; we therefore present adjusted analyses and interpret results cautiously. Due to this imbalance and the limited sample size, no further inter-group comparison of the impact of treatment on renal prognosis was made. Secondly, this was a retrospective single-center design, which limits generalizability. Future multicenter prospective cohort studies are needed to validate these findings. The small sample size resulted in insufficient statistical power; formal sample size calculations should guide future trials. Additionally, the lack of pathological stratification data (MEST-C scores) at baseline limits our ability to predict treatment response based on histological severity. Future studies should incorporate detailed pathological scoring and develop predictive models for treatment outcomes. Thirdly, with the increasing use of oral budesonide in the treatment of IgA nephropathy (KDIGO, 2024; Barratt et al., 2023), the efficacy and safety of combining Telitacicept with Nefecon requires further investigation.

In summary, telitacicept, as monotherapy or combined with immunosuppressive treatment, can significantly reduce proteinuria in patients with IgA nephropathy, resulting in clinical remission of IgA nephropathy in a majority of patients, and has a manageable toxicity profile. In patients with better baseline renal function, telitacicept combined with low-dose corticosteroids may achieve a higher remission rate, though the observed differences may be influenced by baseline characteristics. Telitacicept in combination with even low-dose immunosuppression may still pose a risk of reduced plasma immunoglobulins, and physicians must be vigilant of the risk of infections, with IgG <4 g/L serving as a practical threshold for enhanced monitoring. Prophylactic use of co-trimoxazole is recommended to reduce the risk of infections in patients receiving telitacicept combined with immunosuppressive treatment.

## Data Availability

The raw data supporting the conclusions of this article will be made available by the authors, without undue reservation.
