# Supplementary material for: Efficacy and safety of telitacicept in the treatment of IgA nephropathy: a single-center, real-world study
Source: Front Pharmacol. 2025 Sep 29;16:1642137. doi: 10.3389/fphar.2025.1642137 (PMC12515874; doi:10.3389/fphar.2025.1642137)
Supplement: Supplementary file 1 [file Table1.docx]

**Supplementary Table S1. Detailed pathological data with MEST-C scores**

| **Patient ID** | **Group** | **M Score** | **E Score** | **S Score** | **T Score** | **C Score** | **Total Score** | **Response at Week 24** |
| --- | --- | --- | --- | --- | --- | --- | --- | --- |
| A01 | Group A | 1 | 0 | 1 | 1 | 0 | 3 | PR |
| A02 | Group A | 1 | 1 | 1 | 2 | 1 | 6 | PR |
| A03 | Group A | 0 | 0 | 1 | 1 | 0 | 2 | PR |
| A04 | Group A | 1 | 0 | 0 | 2 | 1 | 4 | No response |
| A05 | Group A | 1 | 1 | 1 | 1 | 0 | 4 | PR |
| A06 | Group A | 0 | 0 | 1 | 0 | 0 | 1 | CR |
| A07 | Group A | 1 | 0 | 1 | 2 | 1 | 5 | No response |
| A08 | Group A | 1 | 1 | 0 | 1 | 0 | 3 | PR |
| B01 | Group B | 1 | 0 | 1 | 0 | 0 | 2 | CR |
| B02 | Group B | 1 | 1 | 1 | 1 | 0 | 4 | PR |
| B03 | Group B | 0 | 0 | 1 | 1 | 0 | 2 | CR |
| B04 | Group B | 1 | 0 | 0 | 0 | 0 | 1 | CR |
| B05 | Group B | 1 | 1 | 1 | 1 | 1 | 5 | PR |
| B06 | Group B | 0 | 0 | 1 | 0 | 0 | 1 | PR |
| B07 | Group B | 1 | 0 | 1 | 1 | 0 | 3 | PR |
| B08 | Group B | 1 | 1 | 0 | 1 | 0 | 3 | PR |
| C01 | Group C | 1 | 0 | 1 | 1 | 0 | 3 | PR |
| C02 | Group C | 1 | 1 | 1 | 0 | 0 | 3 | PR |
| C03 | Group C | 0 | 0 | 1 | 1 | 0 | 2 | CR |
| C04 | Group C | 1 | 1 | 1 | 2 | 1 | 6 | No response |
| C05 | Group C | 1 | 0 | 0 | 1 | 0 | 2 | PR |
| C06 | Group C | 0 | 0 | 1 | 0 | 0 | 1 | PR |
| C07 | Group C | 1 | 1 | 1 | 1 | 0 | 4 | PR |
| C08 | Group C | 1 | 0 | 1 | 1 | 1 | 4 | PR |

**Supplementary Table S2. Detailed follow-up data for patients treated ≥64 weeks**

| **Patient ID** | **Group** | **Baseline 24hUTP (g/d)** | **Week 32** | **Week 40** | **Week 48** | **Week 56** | **Week 64** | **eGFR at Week 64 (mL/min/1.73m²)** | **Adverse Events** |
| --- | --- | --- | --- | --- | --- | --- | --- | --- | --- |
| A03 | Group A | 3.89 | 0.98 | 0.76 | 0.65 | 0.52 | 0.41 | 52.3 | Injection site reaction (n=1) |
| B01 | Group B | 4.56 | 0.52 | 0.38 | 0.29 | 0.24 | 0.18 | 98.7 | None |
| B04 | Group B | 5.23 | 0.48 | 0.31 | 0.26 | 0.22 | 0.19 | 105.2 | Upper respiratory tract infection (n=2) |
| C05 | Group C | 6.12 | 0.89 | 0.62 | 0.48 | 0.35 | 0.28 | 89.4 | Upper respiratory tract infection (n=1) |
